# Potential of methylene blue fluorescence angiography for multiple intraoperative tissue perfusion assessments in plastic and reconstructive surgery: current knowledge and prospects

**DOI:** 10.3389/fsurg.2025.1681731

**Published:** 2025-11-25

**Authors:** Danique J. I. Heuvelings, Lars E. Hillege, Mahdi Al-Taher, Rutger M. Schols

**Affiliations:** 1 Department of Surgery, Maastricht University Medical Center+, Maastricht, Netherlands; 2 NUTRIM School of Nutrition and Translational Research in Metabolism, Maastricht University, Maastricht, Netherlands; 3 GROW—Research Institute for Oncology and Reproduction, Maastricht University Medical Center+, Maastricht, Netherlands; 4 Department of Surgery, Tawam Hospital Al-Ain, Al Ain, United Arab Emirates; 5 Department of Plastic, Reconstructive and Hand Surgery, Maastricht University Medical Center+, Maastricht, Netherlands; 6 Department of Plastic, Reconstructive and Aesthetic Surgery, University Hospital Brussels, Brussels, Belgium; 7 Department of Plastic Surgery, Free University Brussels, Brussels, Belgium

**Keywords:** fluorescence imaging, reconstructive flap surgery, methylene blue, indocyanine green, fluorescence angiography

## Abstract

The expansion of (minimally invasive) surgical techniques has led to the development of advanced tools, including high-definition visual systems and fluorescence-guided surgery (FGS), enhancing surgeons' performance and patient safety. FGS employs near-infrared light and fluorophores like indocyanine green (ICG) for intraoperative imaging to improve anatomical navigation and decision-making. Recently, an increasing number of fluorescence imaging systems have shown capability in visualizing methylene blue (MB), which has an excitation peak at 700 nm, and its potential role in perfusion assessment. A recent study has highlighted MB's advantage over ICG due to its shorter washout time, enabling repeated intraoperative perfusion assessments using a dose of 0.5 mg/kg in a porcine model. This characteristic may be particularly beneficial in plastic and reconstructive surgery involving free (chimeric) flaps, pedicled skin flaps, pedicled intestinal transplants, and mastectomy skin flaps, where perfusion assessment can be critical at multiple stages. Therefore, we highlight some examples of flap reconstructions in this technical note. Important to know, MB is considered safe within the therapeutic doses (<2 mg/kg). However, it is contraindicated in certain patients, including those who are pregnant, have renal insufficiency, are on specific serotonin medications and some rare deficiencies. As dual-wavelength fluorescence imaging systems compatible with both ICG and MB advance, there is growing interest in the potential of MB for (multiple) flap perfusion assessment(s) in plastic and reconstructive surgery, building on promising research in bowel perfusion.

## Introduction

In recent years, the expansion of (minimally invasive) surgical techniques has led to the development of various tools and instruments designed to enhance surgeons' performance, improve patient safety, and reduce the risk of human error (1). Notable among these advancements are high-definition visual systems, as well as fluorescence-guided surgery (FGS) (1, 2). This intraoperative imaging system aids in anatomical navigation during surgery, thereby improving decision-making throughout the procedure (3, 4).

FGS predominantly employs invisible near-infrared light for enhanced visualization (2). Near-infrared fluorescence (NIRF) imaging necessitates the use of a fluorophore in conjunction with an imaging system to detect and measure fluorescence following excitation (5). Fluorophores can be administered locally or systemically, depending on the clinical situation. FGS is most often used for tissue perfusion assessment, lymphography and lymph node detection, delineation of vital **anatomical** structures, and tumor imaging (4). Indocyanine Green (ICG) is the most widely used fluorophore, with most surgical imaging systems supporting its visualization (4). Yet, recent studies have demonstrated that new systems are enhancing their capability to allow for fluorescence imaging with dyes such as Methylene Blue (MB) (6–10). Ideally, camera systems should support dual-wavelength fluorescence imaging, enabling the simultaneous visualization of both MB and ICG (7–9).

There are differences between MB and ICG (Table 1). MB has an excitation and emission wavelength of 668 nm and 688 nm respectively (5, 11). While the technical characteristics of MB pose challenges for visualization compared to ICG which is excited at almost 800 nm, its preferential excretion via the kidneys with minimal hepatic clearance facilitates visualization of the ureters. Besides ureter visualization, the use of MB has also previously been well-documented visualizing thyroid and parathyroid glands, pancreatic neuroendocrine tumors, and breast cancer margin detection (5), and more recently also for bowel perfusion assessment (Supplementary Video S1) (7, 8). The use of MB is promising for plastic and reconstructive surgery, as ICG fluorescence angiography (FA) has already shown it can objectively assess tissue perfusion in flaps, potentially reducing postoperative necrosis risk (12, 13). The objective of this paper is to explore whether MB could be useful for repeated, accurate, and safe intraoperative assessment of flap perfusion during reconstructive procedures, thereby overcoming the limitations associated with ICG's longer washout time and improving real-time surgical decision-making.

**Table 1:** Characterstics of MB and ICG (5, 7, 11).

	Methylene Blue	Indocyanine Green
**Molecular weight**	319.9 g/mol	775.0 g/mol
**Excitation/absorption peak**	665–668 nm	787–789 nm
**Emission wavelength**	686–688 nm	813–819 nm
**Washout time (based on 2.0 mg/kg)**	<1 h	>1 h
**Excretion metabolism**	Removed by the kidneys from circulation to urine (75%) and additionally by the liver	Removed exclusively by the liver from circulation to bile juice

## Idea's: what is the potential of MB for intraoperative tissue perfusion assessments?

A decade ago, Ashitate et al. investigated the role of MB in postoperative flap outcomes in a porcine model (14). They used a dose of 2.0 mg/kg and stated that MB could be administered immediately after flap creation to assess perfusion and predict outcomes on postoperative day 3. However, the further role of MB has not been thoroughly investigated recently. In the recent bowel perfusion assessment study, MB (Proveblue, Provepharm Life Solutions, Marseille, France) was diluted in a sterile phosphate-buffered saline solution to a concentration of 1 mg/mL and administered intravenously with an optimal dose of 0.5 mg/kg using a highly sensitive camera system (7). With this dose, MB offered an advantage over ICG in perfusion assessment due to its shorter washout time. MB was no longer visible 40–50 min after injection in the mesenterial arteries (Supplementary Video S2), whereas ICG did (Supplementary Video S3). This faster washout time allows for a second or even third intraoperative assessment to re-evaluate perfusion status. Although MB showed a stronger correlation between local lactate levels and ingress values, ICG demonstrated higher absolute ingress values (7). However, the research team considered these differences to be negligible, as both dyes provided clear and informative real-time images during the procedure. We believe especially the washout time difference can be useful in reconstructive flap surgery. The most crucial applications of FA in plastic and reconstructive surgery include its use in procedures involving free flap, pedicled flap, mastectomy skin flap and nipple-areolar complex (12).

## Discussion

During free flap reconstruction, perfusion assessment can be performed at different stages of the procedure: e.g., after flap harvest and following the creation of the microvascular anastomosis. However, it is described that perfusion assessment with ICG is typically conducted only once at a single time point (12). Given the larger size of some flaps, NIRF can be conducted across different zones and analyzed accordingly. This often results in the decision to excise a portion of the flap, after which perfusion assessment can be done again. Once the flap is transplanted to the recipient site and the microvascular anastomosis is completed, reassessing perfusion is highly valuable. The rapid clearance of MB offers a significant advantage at this stage, allowing for thorough examination of the transplanted flap even during expedited procedures. In contrast, ICG's longer washout time and tendency for accumulation and interference during reevaluation present significant drawbacks.

Some notable examples of the use of NIRF in pedicled flaps include muscle-sparing transverse rectus abdominis musculocutaneous (TRAM) flaps or fasciocutaneous anterolateral thigh (ALT) flaps. An ALT flap can for example be transferred to an abdominal wall defect in the case of an enterocutaneous fistula (15). Given the often extensive defects and large flaps, it is essential to ensure that the edges do not exhibit ischemia or dehiscence, as these conditions can result in devastating wound complications, such as recurrent fistulas. FA can be initially applied on the leg and subsequently reassessed once the flap is rotated onto the abdomen following tunneling through the groin.

Another example of a pedicled transplant in reconstructive surgery is the intestinal vaginoplasty, which can be carried out as a procedure for vaginal (re)construction. During the operation, a pedicled segment of the intestine is moved downward to form the lining of the neovaginal cavity, typically using a segment from the sigmoid colon or ileum. To achieve sufficient mobility of the segment, some arterial structures may need to be sacrificed, which can negatively impact the blood flow to the segment and, consequently, its viability. NIRF can be utilized to assess the perfusion of the intestinal segment (16, 17). Overall, MB can aid in the assessment of the flap during different stages (e.g., when raising the flap, for perforator selection, after transfer to the recipient site).

During immediate breast reconstruction (IBR, either autologous or alloplastic) it is ideal to conduct perfusion assessment of the mastectomy skin flap and/or nipple-areolar complex at several time points: after the mastectomy and before flap or implant insertion, after flap or implant insertion, and in some cases after the dermis is sutured (18–20). Additionally, in these procedures, it is noted that perfusion assessment using ICG typically occurs at a single timepoint (12). MB may again facilitate multiple intraoperative measurements in a single surgical procedure.

MB is considered safe when used within therapeutic doses of less than 2 mg/kg (11). Therefore, repeated measurements with the proposed lower doses (0.25–0.50 mg/kg) are feasible. IV injection can temporarily interfere with the pulse oximeter's light emission, causing falsely low oxygen saturation readings which return to normal shortly after (5, 11). Rare adverse effects in higher doses include burning sensations, rash, abscess, necrosis, ulceration, nausea, vomiting, abdominal pain, dizziness, hypertension, mental confusion and green urine (5, 21), or cardiovascular events in even more elevated doses (5, 11). Importantly, the use of MB is contraindicated in pregnant patients, or with renal insufficiency, glucose-6-phosphate dehydrogenase (G6PD) deficiency, Heinz body anemia, and selective serotonin(-norepinephrine) reuptake inhibitors (5, 11, 22). An overview of adverse events, contraindications and drug interactions can be found in Table 2.

**Table 2:** Overview of adverse events, contraindications and drug interactions of MB.

Adverse events	Contraindications	Drug interactions
• Bluish-green discoloration of urine • Temporary desaturation in blood • Limb pain or burning sensation following IV administration • Skin: rash, abscess, necrosis, and ulceration • Central nervous system-related symptoms: dizziness, confusion, headaches • In neonates: hyperbilirubinemia, respiratory depression, pulmonary edema, phototoxicity, and hemolytic anemia Only in high doses: • Cardiovascular: cardiac arrhythmias, coronary vasoconstriction, lower cardiac output, decreased renal function, decreased mesenteric blood flow, increased pulmonary vascular pressure • Anaphylaxis (rare)	• Known hypersensitivity reaction • Glucose-6-phosphate dehydrogenase (G6PD) deficiency • Heinz body anemia • Pregnant women • Renal failure	• Serotonergic drugs such as SSRIs, SNRIs, MAOIs, and TCAs due to the MAOI activity

As dual-wavelength fluorescence imaging systems compatible with both ICG and MB continue to develop, there is growing interest in exploring MB's potential role in assessing tissue perfusion within the evolving field of plastic and reconstructive surgery, especially when multiple intraoperative fluorescence assessments are required. Since this Idea and Innovation paper is primarily based on preclinical data, further research is needed to proof this concept in a preclinical model, and additionally support its translation into clinical practice. During these experiments, standardized conditions must be applied to ensure the validity of the conclusions. In the bowel perfusion assessment study (7), the camera was mounted on a custom-made mechanical arm attached to the surgical table, maintaining a fixed distance of 15 cm from the target organ throughout all procedures. Also, to minimize interference from ambient light, the operating room lights were dimmed during imaging. We encourage the use of similar standardized protocols in future studies to allow for reliable comparison of outcomes.

## Data Availability

The original contributions presented in the study are included in the article/Supplementary Material, further inquiries can be directed to the corresponding author.

## Supplementary material

Supplementary Video S1 Intravenous injection of MB (0.5 mg/kg): small bowel perfusion assessment of a female pig. (A) Color image of the surgical field, (B) NIRF image, (C) gradient overlay image, and (D) green overlay image.

Supplementary Video S2 Small bowel perfusion assessment of a female pig 50 min after MB injection. (A) Color image of the surgical field, (B) NIRF image, (C) gradient overlay image, and (D) green overlay image.

Supplementary Video S3 Small bowel perfusion assessment of a female pig 50 min after ICG injection. (A) Color image of the surgical field, (B) NIRF image, (C) gradient overlay image, and (D) green overlay image.
